# Supplementary material for: Metabolomics Insights into Inflammatory Bowel Disease: A Comprehensive Review
Source: Pharmaceuticals (Basel). 2021 Nov 20;14(11):1190. doi: 10.3390/ph14111190 (PMC8625096; doi:10.3390/ph14111190)
Supplement: Supplementary file 1 [file pharmaceuticals-14-01190-s001.zip › pharmaceuticals-1399074-supplementary.pdf]

Supplementary Table S1. Characteristics of metabolomics studies in Inflammatory Bowel Disease.

Authors, year.	Patients	Samples	Method	Results
Bezabeh et al., 2001[14]	21 CD and 26 UC	Colon biopsies	^{1}H NMR	Accuracy to distinguish between CD and UC was 98.6% (diagnostic spectral regions included taurine, lysine, and lipids). Classification accuracy between non-inflamed and inflamed tissues was 97.9%.
Marchesi et al., 2007[52]	10 CD, 10 UC and 13 HC	Faeces	^{1}H NMR	Reduced levels of butyrate, acetate, methylamine, and trimethylamine and elevated quantities of amino acids in CD and UC patients. Lower concentration of glycerol in CD patients. Differences were more marked in CD.
Balasubramanian et al., 2009[15]	20 aUC, 11 qUC,20 aCD and 6 qCD and 26 HC	Colon biopsies	^{1}H NMR	Active IBD patients had lower concentration of amino acids (isoleucine, leucine, valine, alanine, glutamate, and glutamine), membrane components (choline, glycerophosphorylcholine and myo-inositol), lactate and succinate and increased levels of α-glucose. In quiescent patients most of the metabolites were similar to HC.
Williams et al., 2009[46]	86 CD, 60 UC and 60 HC	Urine	^{1}H NMR	Hippurate and 4-cresol sulphate levels were significantly lower and formate significantly higher in CD patients in comparison with UC and HC; same differences in UC vs. HC.
Jansson et al., 2009[53]	8 CD twin pairs and 7 healthy twin pairs	Faeces	Ion cyclotron resonance- Fourier transform mass spectrometry	Differences in the metabolome of patients with colonic CD, ileal CD and HC mainly involving the metabolism or synthesis of amino acids, fatty acids, bile acids and arachidonic acid.
Bjerrum et al., 2010[16]	41 aUC, 33 qUC and 25 HC	Colon biopsies and urine	^{1}H NMR	Higher levels of antioxidants and amino acids in colonic biopsies of active UC in comparison with HC. Significant separation in metabolic profile of colonocytes of HC and active UC. No difference in metabolic profile of colonic biopsies, colonocytes and lymphocytes of HC and quiescent UC. No differences in urine metabolomics in the three groups.
Sharma et al., 2010[17]	12 UC, 9 CD and HC	Colon biopsies	^{1}H NMR	No difference in metabolomics profiles of involved and un-involved colonic mucosa of IBD patients. Un-involved colonic mucosa of IBD patients had higher concentration of amino acids, membrane metabolites, glycolytic products and SCFA and higher level of glucose in comparison with HC.
Ooi et al., 2011[18]	13UC, 21 CD and 17 HC	Colon biopsies and serum	GC-MS	Lower levels of 16 amino acids and molecules involved in the TCA in the colonic biopsies of UC patients. Amino acids and TCA cycle-metabolites in serum were different in UC vs CD or HC
Le Gall et al., 2011[54]	13 UC, 10 IBS and 22 HC	Faeces	^{1}H NMR	NMR spectra were able to distinguish UC and HC. Increase in taurine and cadaverine in UC.
Williams et al., 2012[33]	24 CD, 20 UC and 23 HC	Serum	^{1}H NMR	NMR spectra were able to differentiate between UC, CD and HC with significant predictive accuracy, highlighting differences in lipid and choline metabolism.
Schicho et al., 2012[39]	20 aCD, 20 aUC and 40 HC	Serum, plasma and urine	^{1}H NMR	OPLS-DA models showed high accuracy to distinguish between both UC and CD and HC for all biofluids examined. Discrimination between the two IBD groups was not clear enough in urinary spectra. Alterations in amino acid, energy household, urea cycle, monosaccharides and other metabolites were observed.
Stephens et al., 2013[47]	17 aCD, 13 qCD, 13 aUC, 17 qUC and 60 HC	Urine	^{1}H NMR	NMR spectra were able to differentiate IBD from HC (TCA cycle intermediates, amino acids, and gut microflora metabolites were mainly involved). Comparison of CD and UC patients revealed discrimination, but removal of patients with the surgical intervention confounder revealed that CD could not be discriminated from UC.
Zhang et al., 2013[40]	20 aUC and 19 HC	Serum	^{1}H NMR	Increased 3-hydroxybutyrate, glucose and phenylalanine, but decreased lipid in serum in comparison with HC.

Dawiskiba et al., 2014[41]	16 aCD, 3 qCD, 12 aUC, 12 qUC and 17 HC	Serum and urine	^{1}H NMR	Active IBD had an increase in leucine, isoleucine, 3-hydroxybutyric acid, N-acetylated compounds, acetoacetate, glycine, phenylalanine, lactate and a decrease in creatine, dimethyl sulfone, choline compounds, and histidine compared to HC. Only two metabolites in serum were different between inactive IBD and HC (increased dimethylamine and decreased dimethyl sulfone). In urine, active IBD was associated with a higher concentration of an unknown metabolite with 4-hydroxyphenyl group and a lower citrate, hippurate, trigonelline, taurine, succinate, 2-hydroxyisobutyrate in comparison with HC. Remission was characterized by a higher concentration of acetoacetate and a lower concentration of citrate, hippurate, taurine, succinate, glycine, alanine and formate in comparison with HC.
Bjerrum et al., 2014[55]	31 qCD, 13 aCD, 29 qUC, 19 aUC and 21 HC	Faeces	^{1}H NMR	Significant differences were found in the metabolic profiles between active IBD and controls and between UC and CD, including a range of amino acids, microbiota-related SFCA, and lactate, suggestive of an inflammation-driven malabsorption and dysbiosis.
Hisamatsu et al., 2015[42]	369 qUC	Plasma	HPLC-MS	Plasma amino acid profiles in UC patients in clinical remission can predict the risk of relapse within one year. Decreased histidine level was associated with increased risk of relapse.
Rantalainen et al., 2015[19]	27 aUC, 21 qUC and 15 C	Colonic biopsies	^{1}H NMR	Highest proportion of significant difference in molecular abundances was found between subjects with active UC vs. controls.
Wilson et al., 2015[43]	40 qCD, 33 aCD, 14 qUC, 19 aUC and 373 HC	Plasma	LC-MS/MS	Decreased TMAO levels were detected in IBD compared to a non-IBD population. No difference was observed in active CD versus inactive CD.
Alonso et al., 2016[48]	203 CD, 213 UC and 100 HC	Urine	^{1}H NMR	UC patients had decreased trigonelline, hippurate, methylsuccinate and phenylacetylglycine and increased citrate. CD patients had decreased trigonelline, citrate, hippurate, 3-hydroxyisovalerate and methylsuccinate and increased dimethylglicine, acetate and alanine. Three metabolites (citrate, hippurate, and 3-hydroxyisovalerate) were found to be significantly associated with disease activity in CD. In UC, high disease activity was associated with low levels of urinary hippurate and 3-hydroxyisovaleric acid.
Nikolaus et al., 2017[44]	324 CD, 211 UC and 100 HC	Serum	HPLC and UPLC-MS	Serum levels of tryptophan were significantly lower in patients with IBD than controls, with a stronger reduction in patients with CD. Negative correlation between serum levels of tryptophan and disease activity. The composition of the faecal microbiota associated with serum levels of tryptophan.
Keshteli et al., 2017[45]	20 qUC (6 relapsed within 12 months)	Serum and urine	LC-MS/MS and ^{1}H NMR	Baseline urinary and serum metabolomics profiling of UC patients with or without clinical relapse within 12 months showed a significant difference. Main metabolites responsible for discrimination were trans-aconitate, cystine and acetamide in urine, and 3-hydroxybutyrate, acetoacetate and acetone in serum.
Bjerrum et al., 2017[23]	49 CD, 38 UC and 37 C	Serum	^{1}H NMR	Metabolic profiles were significantly different between active CD/UC and HC, and inactive CD and HC. Metabolites holding differential power belonged primarily to lipids and phospholipids with proatherogenic characteristics and metabolites in the pyruvate metabolism, suggestive of an intense inflammation-driven energy demand. IBD patients not responding to IFX were identified as potentially distinct group based on their metabolic profile, although no applicable response biomarkers could be singled out in this study.

Santoru et al., 2017[56]	82 UC, 50 CD, and 51 HC	Faeces	GC-MS	Various metabolites including biogenic amines, amino acids and lipids were significantly increased in IBD, while others, such as two B-group vitamins, were decreased in IBD compared to HC. This study underlines the potential role of an inter-omics approach in understanding the metabolic pathways involved in IBD.
Probert et al., 2018[26]	40 UC	Plasma	^{1}H NMR	Metabolic profiles of UC active and inactive patients were different. Isoleucine, valine, glucose, and myo-inositol were significantly increased in plasma from individuals with high UCEIS compared to those with low UCEIS, while lipoproteins were all decreased in plasma from high in comparison with low UCEIS. A different metabolite profile, dominated by changes in lysine, histidine, phenylalanine, and tyrosine, distinguished between improvement in UCEIS and worsening (decreased in worsening groups) over 6 months with an accuracy of 74±4%
Murgia et al., 2018[24]	21 qCD, 29 aCD, 39 qUC, 39 aUC and 60 HC	Plasma	LC-MS/MS	Phosphatidylcholines, lysophosphatidylcholines and fatty acids were significantly changed among pathological samples suggesting changes in phospholipase A2 and arachidonic acid metabolic pathways. A decrease in amino acids levels suggests mucosal damage in IBD.
Scoville et al., 2018[25]	15 qCD, 5 aCD, 17 qUC, 3 aUC and 20 HC	Serum	HILIC/UPLC-MS/MS	Most of the alterations occurred in lipid-, amino acid-, and energy-related metabolites. Comparing only CD and control subjects revealed 286 significantly altered metabolites, whereas comparing UC and control subjects revealed only 5 significantly altered metabolites. Hierarchical clustering using significant metabolites separated CD from UC and control subjects.
Keshteli et al., 2018[20]	38 CD (previous ileocolonic resection)	Ileal biopsies	LC-MS/MS and ^{1}H NMR	Endoscopic recurrence was associated with increased concentration of urinary levoglucosan. Endoscopic postoperative recurrence was positively correlated with levoglucosan and propylene glycol levels.
Sofia et al., 2018[28]	99 UC	Serum	HPLC and GC-MS	Increasing Kynurenic acid/tryptophan ratio was closely associated with endoscopic inflammation and predictive of disease outcomes. These findings support the role of the kynurenine pathway in regulating mucosal inflammation in UC.
Piestansky et al., 2019[49]	13 CD and 10 HC	Urine	CE-MS/MS	Significant differences were observed in amino acids levels between IBD patients undergoing thiopurine treatment, which could indicate that amino acids analysis could be a valuable tool for the study of mechanism of the IBD treatment by thiopurines.
Lai et al., 2019[29]	10 aCD, 10 qCD and 10 HC	Serum	LC-MS/MS	Pathway perturbations ranging from energy metabolism (e.g., β-oxidation of fatty acids) to signalling cascades of lipids (e.g., DHA) and amino acids (e.g., L-tryptophan) were detected in CD patients. Importantly, an integral role of gut microbiota in the pathogenesis of CD was highlighted.
Keshteli et al., 2019[50]	31 IBS, 53 qUC and 21 HC	Urine	LC-MS/MS and GC-MS	Increase in lactic acid, proline, oxoglutaric acid, ethylmalonic acid, glutamic acid, 3-hydroxyisovaleric acid, citrulline, hydroxyphenylacetic acid and adipic acid; and a decrease in glutamine, histidine, lysine, phenylalanine and Sumiki's acid, in UC compared to IBS.
Lloyd-Price et al., 2019[57]	67 CD, 38 UC and 27 non-IBD	Faeces	LC-MS	Metabolite pools were less diverse in individuals with IBD. The smaller number of compounds that were more abundant in patients with IBD included polyunsaturated fatty acids such as adrenate and arachidonate. Pantothenate and nicotinate (vitamins B5 and B3, respectively) were particularly depleted in the IBD's gut.
Diab et al., 2019[21]	18 treatment naïve UC, 10 qUC and 14 HC	Colonic biopsies	GC-MS and UPLC-MS	Several pathways were highly perturbed, ranging from amino acid metabolism (such as tryptophan metabolism, and alanine, aspartate and glutamate metabolism) to antioxidant defence pathway (glutathione pathway). The pathway analysis revealed a

				disruption in the long- and short-chain fatty acid metabolism, namely linoleic metabolism and butyrate metabolism.
Franzosa et al., 2019[58]	68 CD, 53 UC and 34 HC	Faeces	LC-MS	Identified 122 robust associations between differently abundant species and well-characterized differently abundant metabolites, indicating possible mechanistic relationships that are perturbed in IBD.
Sun et al., 2019[30]	23 aUC, 25 qUC and 30 C	Faeces and plasma	LC-MS	TMAO was higher in UC patients, but no statistical difference was found between active and inactive UC. Sphingosine 1-phosphate was increased in UC, which could become a new target for UC treatment.
Roda et al., 2019[31]	40 CD, 40 UC	Serum	HPLC-MS/MS	PCA allowed the separation into two clusters within CD (biologic-free patients and patients treated with anti-TNF alpha drugs and HC) but not in UC.
Krzystek-Korpacka et al., 2020[32]	40 aCD, 12 qCD, 33 aUC, 15 qUC, 18 IBS and 40 HC	Serum	LC-MS/MS	Dimethylarginine decreased in active CD compared to controls and active UC. Citrulline and dimethylamine increased in active CD and active UC compared to controls.
Di Giovanni et al., 2020[34]	35 CD and 33 HC	Serum	GC-MS	Several metabolites were different in CD and HC, including threonic acid, aspartic acid, glutamic acid, xylose, methionine, 2-hydroxybutyric acid, 1,5-anhydroglucitol, citric acid and galactose. Several metabolites are potential biomarkers of disease activity within CD patients: capric acid, erythritol/threitol, myristic acid, glucose and lauric acid.
Borren et al., 2020[35]	106 qCD and i60 UC (55% with fatigue)	Serum	LC-MS/MS	Patients with fatigue had lower levels of methionine, tryptophan, proline and sarcosine. Faecal samples from patients with fatigue had a less diverse gut microbiome. The fatigue-like microbiome was associated with fatigue scales and correlated with progressive depletion of metabolites from serum samples.
Ding et al., 2020[36]	76 CD, 23 UC and 13 HC	Serum, urine and faeces	HILIC/UPLC-MS	Histidine and cysteine in serum and urine were biomarkers of response. Bile acid profiling identified primary bile acids to be associated with non-response to anti-TNF therapy, with higher levels of phase 2 conjugates in non-responders. Receiver operating characteristics curves for treatment response demonstrated 0.94+/-0.10 (faecal lipid), 0.81+/-0.17 (faecal bile acid) and 0.74+/-0.15 (serum bile acid) predictive ability for anti-TNF response in CD.
Borren et al., 2020[37]	108 qCD and 56 qUC	Serum	LC-MS	4 metabolomics markers (propionyl-L-carnitine, carnitine, sarcosine, and sorbitol) were associated with relapse in multivariable models. Proteomic and metabolomics risk scores independently predicted relapse with a combined area under the curve of 0.83. A high proteomic risk score (odds ratio = 9.11; 95% confidence interval, 1.90-43.61) or metabolomics risk score (odds ratio = 5.79; 95% confidence interval, 1.24-27.11) independently predicted a higher risk of relapse over 2 years.
Lins et al., 2020[59]	10 CD, 11 UC and 15 HC	Faeces	^{1}H NMR	Lactate, succinate, alanine, and tyrosine, in the CD faecal samples, and leucine, alanine, and tyrosine in the UC faecal samples. All the amino acids presented positive covariance for disease correlation.
Tefas et al., 2020[38]	5 aCD, 17 aUC and 24 HC	Plasma	UPLC-MS	7 metabolites were significantly altered in IBD patients compared to HC.
Santoru et al., 2021[22]	50 CD, 82 UC and 51 HC	Plasma and biopsies	GC-MS	Multivariate statistical analysis of the identified metabolites in CD and UC showed changes in energy metabolism, and lactic acid and ornithine were altered in both plasma and colon biopsies. Metabolic changes were evidenced between the normal ileum and colon tissues. These differences disappeared when inflamed ileum and colon tissues were compared, suggesting a common metabolism.

Notararigo et al., 2021[27]	18 qCD, 9 qUC and 10 HC	Blood and serum	^{1}H NMR	Higher levels of homoserine-methionine and isobutyrate were identified as biomarkers of ileocolic CD. For UC, lower levels of creatine-creatinine, proline, and tryptophan were found reflecting a deficit in the absorption of essential amino acids in the gut.
Fang et al., 2021[51]	79 CD and 50 UC	Faeces	LC-MS/MS	Several metabolites are differentially abundant in individuals with prior surgery; most of these were bile acids. Both tyrosine and glutamic acid are less abundant in subjects with surgery.

CD, Crohn's disease; aCD, active Crohn's disease; qCD, quiescent Crohn's disease; UC; ulcerative colitis; aUC; active ulcerative colitis; qUC; quiescent ulcerative colitis; IBD; inflammatory bowel disease; HC, healthy controls; HPLC, high performance liquid chromatography; ^{1}H NMR; proton magnetic resonance spectroscopy; IBS, irritable bowel syndrome; GC-MS, gas chromatography/mass spectrometry LC-MS/MS, liquid chromatography tandem mass spectrometry; UPLC, ultra-performance liquid chromatography; HILIC, hydrophilic interaction liquid chromatography; CE-MS/MS, capillary electrophoresis-tandem mass spectrometry; TCA, tricarboxylic acid; TMAO, Trimethylamine N-oxide; IFX, infliximab; UCEIS, UC endoscopic index of severity.
